# Transcriptome analysis of tomato plants following salicylic acid-induced immunity against *Clavibacter michiganensis* ssp. *michiganensis*

**DOI:** 10.5511/plantbiotechnology.23.0711a

**Published:** 2023-12-25

**Authors:** Naoki Yokotani, Yoshinori Hasegawa, Yusuke Kouzai, Hideki Hirakawa, Sachiko Isobe

**Affiliations:** 1 Kazusa DNA Research Institute, 2-6-7 Kazusa-Kamatari, Kisarazu, Chiba 292-0818, Japan; 2 Bioproductivity Informatics Research Team, RIKEN Center for Sustainable Resource Science, 1-7-22 Suehiro-cho, Tsurumi, Yokohama, Kanagawa 230-0045, Japan

**Keywords:** *Clavibacter michiganensis* ssp. *michiganensis*, plant immunity, salicylic acid, tomato, transcriptome

## Abstract

Salicylic acid (SA) is known to be involved in the immunity against *Clavibacter michiganensis* ssp. *michiganensis* (*Cmm*) that causes bacterial canker in tomato. To identify the candidate genes associated with SA-inducible *Cmm* resistance, transcriptome analysis was conducted via RNA sequencing in tomato plants treated with SA. SA treatment upregulated various defense-associated genes, such as PR and GST genes, in tomato cotyledons. A comparison of SA- and *Cmm*-responsive genes revealed that both SA treatment and *Cmm* infection commonly upregulated a large number of genes. Gene Ontology (GO) analysis indicated that the GO terms associated with plant immunity were over-represented in both SA- and *Cmm*-induced genes. The genes commonly downregulated by both SA treatment and *Cmm* infection were associated with the cell cycle and may be involved in growth and immunity trade-off through cell division. After SA treatment, several proteins that were predicted to play a role in immune signaling, such as resistance gene analogs, Ca^2+^ sensors, and WRKY transcription factors, were transcriptionally upregulated. The W-box element, which was targeted by WRKYs, was over-represented in the promoter regions of genes upregulated by both SA treatment and *Cmm* infection, supporting the speculation that WRKYs are important for the SA-mediated immunity against *Cmm*. Prediction of protein–protein interactions suggested that genes encoding receptor-like kinases and EF-hand proteins play an important role in immune signaling. Thus, various candidate genes involved in SA-inducible *Cmm* resistance were identified.

## Introduction

Plant disease is a serious problem for crop production, and understanding plant immune mechanisms is important to control pathogens and breed resistant cultivars. Plants have two immune systems, namely pattern-triggered immunity (PTI) and effector-triggered immunity (ETI), through which they recognize pathogens and elicit defense responses (Hou et al. 2019; Saijo et al. 2018; Schwessinger and Ronald 2012). PTI is induced when structurally conserved molecules derived from pathogens, known as microbe- or pathogen-associated molecular patterns (MAMPs/PAMPs), are recognized by pattern recognition receptors (Tang et al. 2017). ETI is induced when pathogen-derived specific effectors are recognized by resistance (*R*) gene products. The rapid and strong expression of defense-associated genes suppresses the infection completely (Schwessinger and Ronald 2012). For example, ETI is induced when pathogen effector proteins (encoded by *AVR*) are recognized by R gene products, such as receptor-like kinases (RLKs), nucleotide-binding sites (NBSs), receptor-like proteins (RLPs), and transmembrane coiled-coils (TM-CC) (Li et al. 2016). The products of the R gene and its homologs with unknown function are referred to as resistance gene analogs (RGAs). Some RLKs are known to exhibit functions other than ETI, and others play important roles in PTI as receptors for MAMPs/PAMPs (Tang et al. 2017). Kinase cascades composed of protein kinases, such as mitogen-activated protein kinase and calcium-dependent protein kinases (CDPKs), are significantly involved in the transduction of pathogen-derived signals to receptor complexes (Tena et al. 2011; Zhou and Zhang 2020). Disease signals regulate the expression of defense-related genes, including PR genes via transcription factors (TFs) such as WRKYs (Ruston et al. 2010; Tsuda and Somssich 2015). Furthermore, in plant immunity, reactive oxygen species, Ca^2+^, and phytohormones play a role in long-range transmission, enhancement, or suppression of secondary signals to distant sites (Saijo et al. 2018).

Tomato (*Solanum lycopersicum*), one of the most important fruit crops worldwide, is targeted by *Clavibacter michiganensis* ssp. *michiganensis* (*Cmm*), which is a gram-positive bacterium that causes bacterial canker (Eichenlaub and Gartemann 2011; Sen et al. 2015). As only a limited number of cultivars are resistant to *Cmm* and few pesticides have shown inhibitory effects against it, it is important to understand the host’s response to control the spread of bacterial canker. Only a few serious gram-positive bacteria are known to severely affect plant production; therefore, the tomato–*Cmm* pathosystem is optimal to study the interaction between plants and gram-positive bacteria. To understand the immune mechanism against *Cmm* in tomato, we used RNA sequencing (RNA-seq) to analyze the host transcriptome in cotyledons after infection (Yokotani et al. 2021). Results showed that the expression of PR genes, RGAs, and genes encoding TFs (such as WRKYs, NACs, and CBP60s) was upregulated after *Cmm* infection. Similarly, other studies have identified candidate genes involved in PTI against *Cmm* (Yokotani et al. 2021). Increased expression of PR, RLK, and ethylene synthesis genes has been observed in tomato stems after *Cmm* infection (Balaji et al. 2008). In contrast, decreased expression of photosynthesis-related genes after *Cmm* infection in tomato stems has been shown to be potentially associated with immunity (Tsitsekian et al. 2021). Transcriptome comparisons between resistant and susceptible tomato species have identified a number of candidate genes involved in immunity (Basim et al. 2021; Lara-Ávila et al. 2012).

Salicylic acid (SA) is a β-hydroxy acid that functions as a plant hormone and is involved in immunity and stress response (Robert-Seilaniantz et al. 2011). We have previously demonstrated that tomato genes orthologous to the *Arabidopsis* genes *EDS1*, *EDS5*, and *PAD4*, which are responsible for SA biosynthesis, were upregulated after *Cmm* infection (Yokotani et al. 2021). Our previous study results also showed that *Cmm* infection stimulated SA accumulation in tomato cotyledons. Furthermore, the exogenous application of SA improved resistance to *Cmm* in tomato seedlings (Yokotani et al. 2021). Similarly, the exogenous application of benzothiadiazole (BTH), a functional analog of SA, was shown to induce resistance to *Cmm* in tomato (Soylu et al. 2003). Therefore, the activation of the SA signaling pathway could protect tomato plants from this bacterium. To elucidate the role of SA in the immunity against *Cmm* in tomato, it is necessary to identify the genes regulated by SA. Several studies have revealed that the exogenous application of SA analogs in plants induces the expression of PR genes, which are often used as molecular markers to identify defense responses (van Loon et al. 2006). Previous microarray analyses have demonstrated that the expression of PR genes, including acidic chitinase and *PR1*, is upregulated after treating tomato leaves with BTH (Zuluaga et al. 2013). The application of exogenous 1,2-benzisothiazol-3(2H)-one1,1-dioxide, which could be converted into SA in plants, was also shown to induce the expression of *PR1*, *PR2*, and *PR5* (Kusajima et al. 2017). In our previous study, we showed that SA treatment induced the expression of numerous WRKY genes, which are involved in the regulation of PR genes (Yokotani et al. 2021). However, only a few studies have been conducted on the effect of native SA on tomato immunity.

As complete tomato genome sequencing was completed in 2012, the motif and function of tomato genes have been annotated (Tomato Genome Consortium 2012). Recently, several tomato genome databases, which include information such as gene expression, gene function, and protein motifs, have been established (Kudo et al. 2017; Menda et al. 2013). Extensive databases specifying the motifs and functions of proteins are also available. Using this information, the current study analyzed RNA-seq data obtained from SA-treated tomato plants to predict the genes involved in immunity against *Cmm*.

## Materials and methods

### Plants and pathogens

Tomato (*Solanum lycopersicum*) plants of the cultivar ‘Moneymaker’ (accession no. TOMJPF00002) were provided by the University of Tsukuba, Tsukuba Plant Innovation Research Center, through the National Bio-Resource Project of the AMED, Tsukuba, Japan. The *Cmm* ssp. *michiganensis* virulent strain MAFF301040 isolated in Tokyo, Japan in 1963 was provided by the MAFF Genebank of the National Agriculture and Food Research Organization.

### SA treatment

Tomato seedlings were cultivated in a growth chamber containing soil under 16 h of light at 25°C for 10 days before SA treatment. The seedlings were transplanted into soil moistened with water containing 1 mM SA and 0.1% (vol/vol) DMSO and were allowed to grow for 24 h. Induced resistance to *Cmm* and upregulation of WRKY genes are observed under these conditions (Yokotani et al. 2021). Soil moistened with water containing 0.1% (vol/vol) DMSO was used as the control.

### *Cmm* infection in tomato cotyledons

The *Cmm* culture (1×10^7^ cfu ml^−1^) was resuspended in infiltration buffer with 10 mM MES, 10 mM MgSO_4_, and 0.02% (vol/vol) Silwet L-77. The cotyledons of 10-day-old seedlings were dipped into the bacterial suspension present in closed conical tubes and were infiltrated by pressurization with a syringe. After washing the surface of the cotyledons with water, the seedlings were transplanted into soil and cultured under high humidity conditions for 7 days. Evident disease symptoms were observed under these conditions (Yokotani et al. 2021).

### RNA isolation and RNA-seq analysis

In this study, the cotyledons subjected to SA treatment and *Cmm* infection, as described above, were used for RNA-seq analysis. Total RNA was isolated from cotyledons using Sepasol-RNA I Super kit (Nacalai-Tesque, Kyoto, Japan). The concentration and quality of RNA were determined using Qubit fluorometer (Thermo Fisher Scientific, MA, USA) and Agilent 2100 bioanalyzer, respectively. The purified total RNA (250 ng) was used for RNA library preparation, according to the instructions of Illumina’s QuantSeq 3′ mRNA-seq library preparation kit (Lexogen, Vienna, Austria). The RNA libraries were sequenced using the Illumina NextSeq 500 system with 75-nt-long reads. Prior to mapping, the raw reads were subjected to adapter removal and read quality trimming. Each read was mapped to the tomato reference genome ITAG4.0 using CLC Genomics Workbench v22 (QIAGEN, Hilden, Germany) with default settings.

RNA-seq analysis was performed in triplicate and the count per million mapped reads (CPM) was used as the transcript level. After log2 transformation of CPM+1, the genes whose expression was significantly altered were selected via one-way analysis of variance followed by false discovery rate (FDR) analysis (FDR<0.3). Genes whose log2 transformed CPM+1 value in SA-treated cotyledons differed by >1 compared with that in DMSO-treated cotyledons were considered to be altered by SA treatment. Genes whose log2 transformed CPM+1 value in *Cmm*-inoculated cotyledons differed by >1 compared with that in buffer-treated cotyledons were considered to be altered by *Cmm* infection.

### Gene Ontology (GO) and Kyoto Encyclopedia of Genes and Genomes (KEGG) enrichment analyses

The protein sequences of ITAG4.0 were functionally annotated using DIAMOND searches (Buchfink et al. 2015) with a more sensitive mode against UniProtKB (Swiss-Prot+TrEMBL; https://www.uniprot.org). GO terms were assigned to each gene using Blast2GO (Conesa et al. 2005) based on similarity searches. Candidates for disease RGAs encoding NBSs, RLPs, TM-CCs, and RLKs were searched via RGAugury (Li et al. 2016). KEGG annotations were obtained from the TOMATOMICS database (Kudo et al. 2017).

### Identification of protein domains using the Pfam database

Tomato ITAG4.0 proteins were functionally annotated via a Pfam domain search. The protein sequences were scanned using the hmmscan function of HMMER program (version 3.3.2, Finn et al. 2011) based on a hidden Markov model of the Pfam database (Pfam34.0, downloaded in May 2021; Mistry et al. 2021). The e-value cutoff was set at 1e−10.

### Promoter analysis

The *cis*-element sequences targeted by WRKYs, HSFs, and CBP60s, which were transcriptionally upregulated by SA treatment or *Cmm* infection, were obtained from previous studies (Busch et al. 2005; Maleck et al. 2000; Sun et al. 2015). Extraction of the 500-bp upstream sequence of each gene based on the SL4.0 genome and ITAG4.0 gene sequences as well as *cis*-element search were performed using “Biostrings” function in R. Hypergeometric enrichment analysis was conducted using “phyper” function in R.

### Prediction of protein–protein interactions

Protein–protein interactions were predicted based on the PTIR database (Yue et al. 2016). The High_quality_0.5 dataset was used in this analysis. Network analysis of genes that were upregulated after SA treatment or *Cmm* infection was conducted using “igraph” packages in R. Proteins that interacted only with themselves were excluded from the network.

## Results

### Transcriptome profiling of tomato after exogenous SA treatment and *Cmm* infection

**Figure 1:**
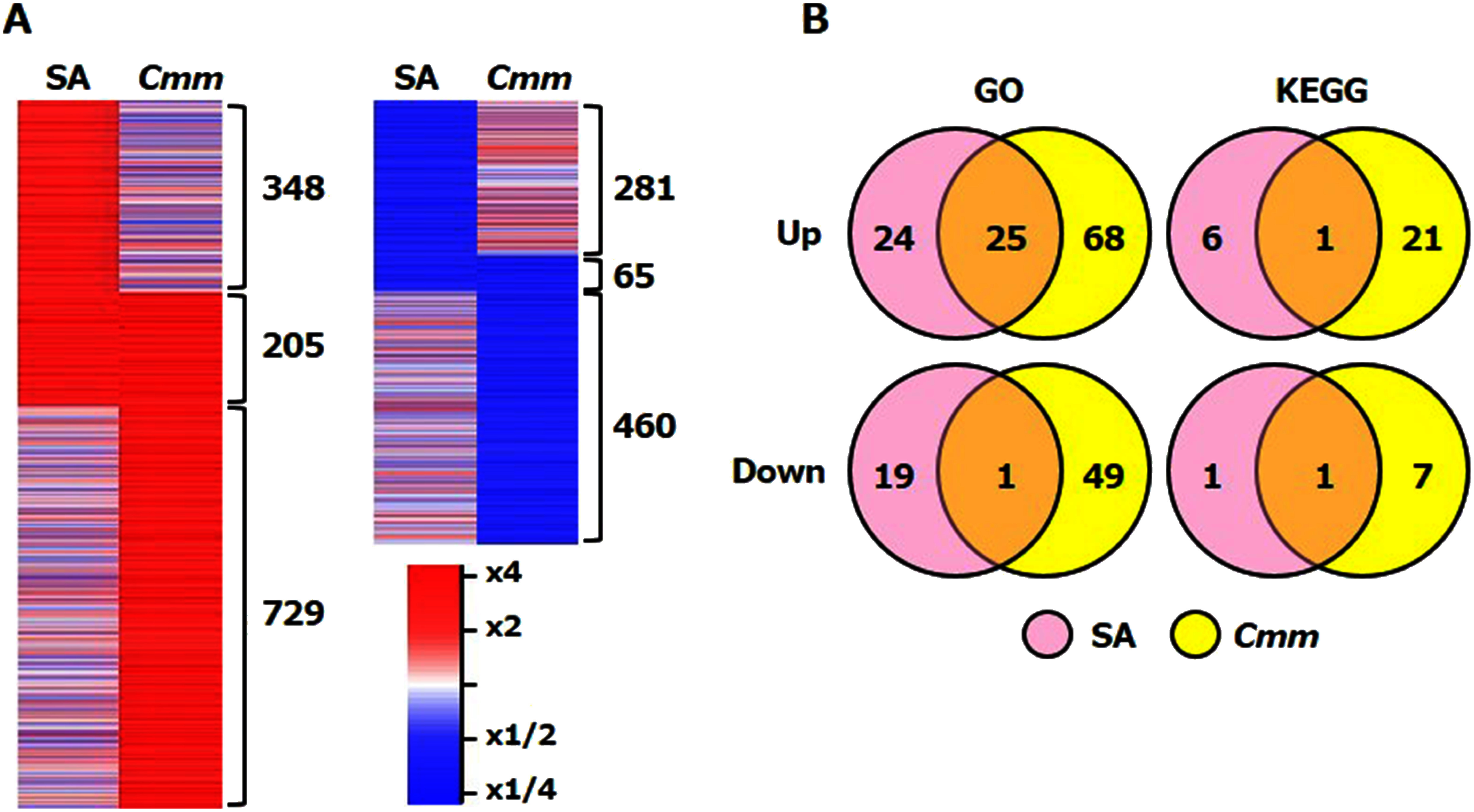
Figure 1. GO and KEGG enrichment analyses and expression of genes altered after SA treatment or *Cmm* infection. (A) Heatmap of genes upregulated (left) and downregulated (right) after SA treatment or *Cmm* infection. The list of genes is included in Supplementary Figure S1. (B) GO and KEGG enrichment analyses of genes upregulated and downregulated after SA treatment or *Cmm* infection. The over-represented GO terms and KEGG pathways in genes altered by SA treatment or *Cmm* infection are listed in Supplementary Tables S3 and S4.

The obtained RNA-seq data were deposited in the DDBJ Sequence Read Archive at the DNA Data Bank of Japan (http://trace.ddbj.nig.ac.jp/dra) under accession numbers DRR463155–463166 (BioProject; PRJDB15775). The analysis generated 1.8–2.2 million raw reads for each sample, and 69.4%–80.0% of them were correctly mapped to the *S. lycopersicum* reference genome (SL4.0) and *S. lycopersicum* gene annotation database (ITAG4.0) of the International Tomato Annotation Group, which contained 34,075 annotated genes (Supplementary Table S1). The mean CPM values in the samples are shown in Supplementary Table S2. In this study, after SA treatment and *Cmm* infection, 553 and 934 genes showed a 2-fold increase in upregulation, whereas 346 and 525 genes exhibited the same increase in downregulation, respectively. A total of 205 and 65 genes were commonly up- and downregulated, respectively, after both interventions (Figure 1A and Supplementary Figure S1).

### Enrichment of GO and KEGG pathways in SA- and *Cmm*-responsive genes

In total, 49 GO terms and 7 KEGG pathways were over-represented in the genes upregulated after SA treatment, whereas 93 GO terms and 22 KEGG pathways were over-represented in those upregulated after *Cmm* infection (Figure 1B, Supplementary Table S3). Overall, 25 GO terms and 1 KEGG pathway were over-represented after both interventions (Figure 1B). These included immune-related GO terms, such as defense response (GO:0006952) and regulation of systemic acquired resistance (GO:0010112). Moreover, GO terms associated with response to external signaling, including the cell surface receptor signaling pathway (GO:0007166), regulation of response to external stimuli (GO:0032101), recognition of pollen (GO:0048544), signaling receptor activity (GO:0038023), hormone-mediated signaling pathway (GO:0009755), and calcium ion binding (GO:0005509), were over-represented. Over-representation was also observed for two GO terms and one KEGG pathway associated with glutathione metabolism, i.e., glutathione metabolic process (GO:0006749), glutathione transferase activity (GO:0004364), and glutathione S-transferase (K00799).

In total, 20 GO terms and 2 KEGG pathways were over-represented in the genes downregulated after SA treatment, whereas 50 GO terms and 8 KEGG pathways were over-represented in those downregulated after *Cmm* infection (Figure 1B, Supplementary Table S4). Overall, only one GO term, i.e., G1/S transition of the mitotic cell cycle (GO:0000082), and one KEGG pathway, i.e., cyclin D3, plant (K14505), were over-represented after both interventions (Figure 1B, Supplementary Table S4).

### Expression of genes involved in the defense response after SA treatment

The expression of 13 and 34 PR genes in tomato cotyledons was transcriptionally upregulated after SA treatment and *Cmm* infection, respectively (Table 1). Among them, seven PR genes were commonly upregulated after both interventions. The hypergeometric enrichment test demonstrated that the PR gene homologs were significantly over-represented in the genes upregulated after both SA treatment and *Cmm* infection (*p*<0.05, hypergeometric distribution test). In the tomato genome, 74 genes encoding protein-carrying domains, such as Pfam PF00043.27, PF13410.8, PF17171.6, PF02798.22, PF13409.8, PF13417.8, or PF17172.6, were identified and considered as GSTs. The expression of 16 and 12 GST genes was transcriptionally upregulated after SA treatment and *Cmm* infection, respectively. Among them, five genes were commonly upregulated after both interventions. GST genes were significantly over-represented after both SA treatment and *Cmm* infection (*p*<0.05).

**Table 1:** Table 1. Defense-associated genes upregulated after SA treatment or *Cmm* infection.

Family	SA		*Cmm*		Common
	Number	*p*-value	Number	*p*-value	
PRs	13	0.002*	34	<0.001*	7
GST	16	<0.001*	12	<0.001*	5
RLK	23	<0.001*	46	<0.001*	13
NBS	12	<0.001*	9	0.205	8
RLP	7	<0.001*	12	<0.001*	6
TM-CC	4	0.217	10	0.007*	3
Calreticulin	2	0.003*	3	<0.001*	2
EF-hand	6	0.021*	21	<0.001*	4
WRKY	9	<0.001*	11	<0.001*	8
HSF	3	0.008*	2	0.159	1
CBP60	1	0.178	4	<0.001*	1

*Significantly enriched gene family, as indicated by the hypergeometric distribution test (*p*<0.05).

After SA treatment, 46 RGAs, including 23 RLKs, 12 NBSs, 7 RLPs, and 4 TM-CCs, were transcriptionally upregulated (Table 1). Among them, RLKs, NBSs, and RLPs were significantly over-represented (*p*<0.05). After *Cmm* infection, the expression of 77 RGAs, including 9 NBSs, 46 RLKs, 12 RLPs, and 10 TM-CCs, was upregulated. Among them, RLKs, RLPs, and TM-CCs were significantly over-represented (*p*<0.05). After both SA treatment and *Cmm* infection, 13 RLKs, 8 NBSs, 6 RLPs, and 3 TM-CCs were commonly upregulated after both treatment and infection.

Various Ca^2+^ sensors have been reported in plants (Dodd et al. 2010). In this study, genes encoding calreticulin and EF-hand-containing proteins were upregulated by SA treatment or *Cmm* infection (Supplementary Table S2). Five calreticulin genes were identified to encode proteins containing the Pfam domain PF00262.20 in the tomato genome (data not shown). Two and three of these calreticulin genes were induced after SA treatment and *Cmm* infection, respectively, and two of them were commonly upregulated after both interventions. These calreticulin genes were significantly over-represented in both SA- and *Cmm*-responsive genes (Table 1). A total of 132 EF-hand proteins were identified to contain domains such as Pfam PF00036.34, PF17958.3, PF12763.9, PF13202.8, PF13405.8, PF13499.8, PF13833.8, or PF14658.8 in the tomato genome. The expression of 6 and 21 EF-hand genes was transcriptionally upregulated after SA treatment and *Cmm* infection, respectively. Among these genes, four were commonly upregulated after both interventions. EF-hand genes were significantly over-represented after both SA treatment and *Cmm* infection (Table 1). Previous studies (Munir et al. 2016; Wang et al. 2016; Zhao et al. 2013) have shown that proteins with EF-hand motifs induced by SA treatment or *Cmm* infection included 1 calmodulin (SlCaM2), 12 calmodulin-like proteins (SlCML1, 3, 18, 29, 31, 34, 35, 37, 38, 39, 44, and 51), and 6 CDPKs (SlCDPK10, 11, 12, 18, 27, and 29).

Various TF gene families were upregulated after SA treatment or *Cmm* infection. Among them, WRKYs, HSFs, and CBP60s were significantly over-represented (*p*<0.05, hypergeometric distribution test) (Table 1). A total of 9 and 11 WRKY genes were upregulated after SA treatment and *Cmm* infection, respectively; among them, eight WRKY genes were commonly upregulated after both interventions (Table 1). The expression of three and two HSF genes was upregulated after SA treatment and *Cmm* infection, respectively, and one gene was commonly upregulated after both interventions. Four CBP60 genes were upregulated after *Cmm* infection, and two of them were also upregulated after SA treatment (Table 1).

### Over-representation of immune-related *cis*-elements in the promoters of SA- and *Cmm*-responsive genes

**Figure 2:**
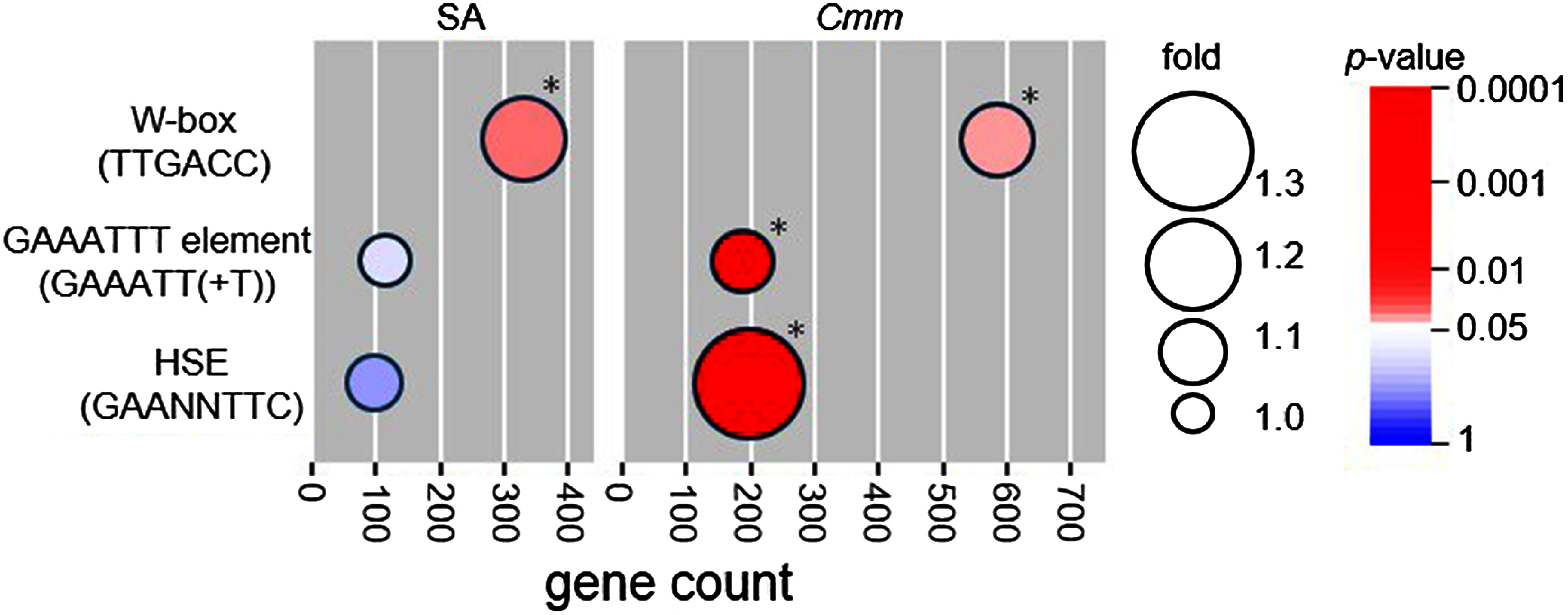
Figure 2. Enrichment analysis of *cis*-elements targeted by WRKYs (W-box), HSFs (HSE), and CBP60s (GAAATTT element). Asterisks indicate *cis*-elements significantly over-represented in the upstream region of genes that were upregulated after SA treatment or *Cmm* infection (as indicated via hypergeometric analysis) (*p*<0.05).

After SA treatment or *Cmm* infection, three TF families, i.e., WRKY, HSF, and CBP60, were significantly over-represented. WRKYs, HSFs, and CBP60s have been reported to bind to the specific W-box (TTGACC), HSE (GAANNTTC), and GAAATTT (GAAATT(+T)) sequences, respectively (Busch et al. 2005; Maleck et al. 2000; Sun et al. 2015). Among 34,075 genes in the SL4.0 and ITAG4.0 tomato genomes, the abovementioned 3 TF families were found within 500-bp upstream regions of 6,093, 19,571, and 5,673 genes, respectively. The number of genes induced by SA treatment and *Cmm* infection containing W-box, HSE, or GAAATTT elements in their upstream regions, and the results of the hypergeometric distribution test are shown in Figure 2. The W-box sequence was over-represented in the promoter region of both SA- and *Cmm*-responsive genes. The GAAATTT and HSE sequences were over-represented in the promoter region of *Cmm*-responsive genes, but not in that of SA-responsive genes.

### Predicted interaction networks of proteins encoded by SA- and *Cmm*-responsive genes

**Figure 3:**
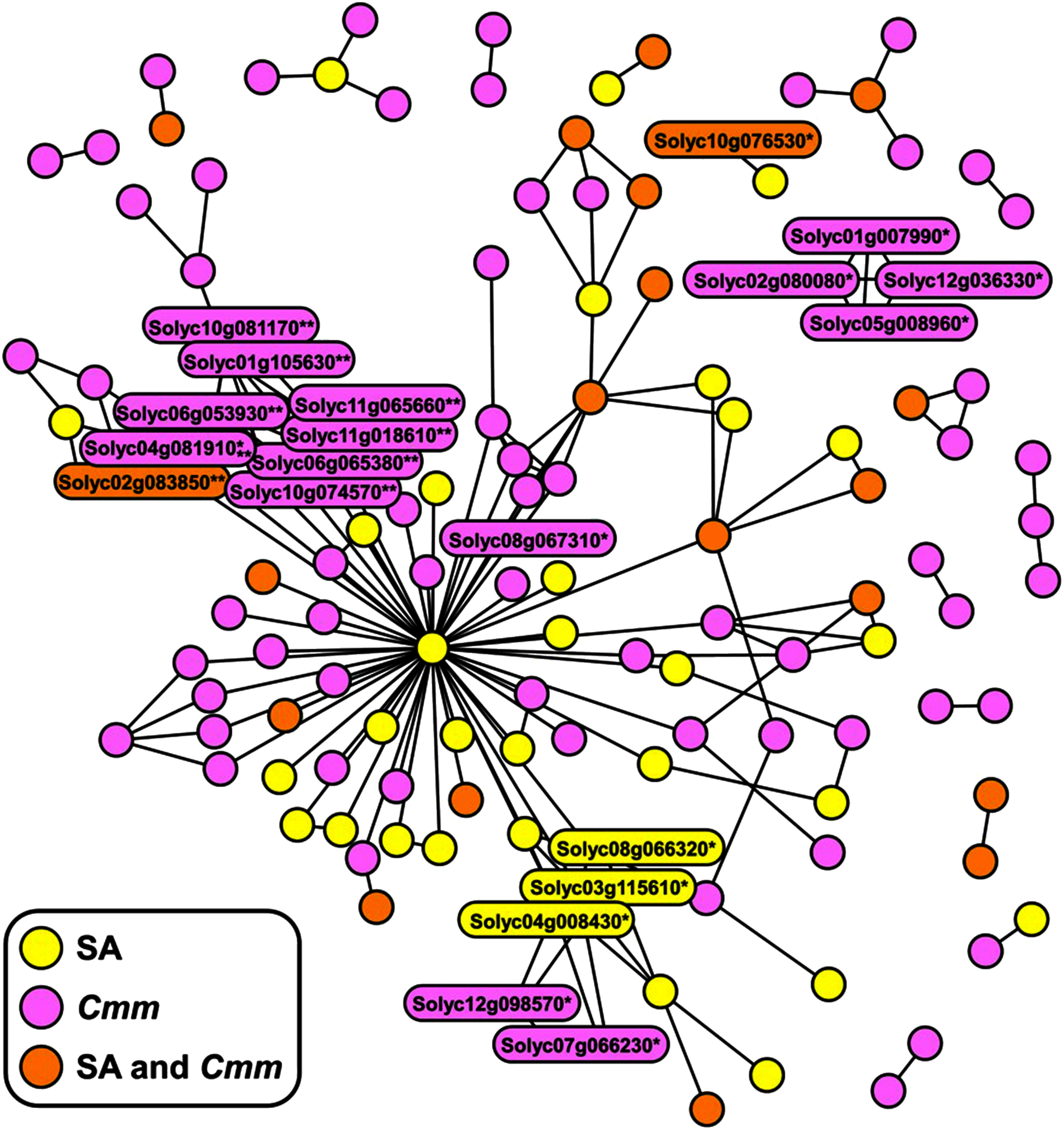
Figure 3. Protein–protein interactome network of genes upregulated after SA treatment or *Cmm* infection. Network analysis was conducted using the High-quality_0.5 database. Genes encoding RLKs and EF-hand proteins are indicated by single and double asterisks, respectively. All genes included in the network are shown in Supplementary Figure S2.

Among 1,282 genes induced by SA treatment or *Cmm* infection, 145 were identified in the protein–protein interaction High_quality_5.0 dataset (Yue et al. 2016). Network analysis generated 177 edges and divided 131 proteins into 17 independent clusters (Figure 3 and Supplementary Figure S2, Supplementary Table S5). The protein with the highest number of edges was Solyc10g086410 (LEHSC270), with 54 edges. A total of 12 RLKs were included in the network, 10 of which had ≥3 edges and 4 had ≥6 edges. Moreover, 9 EF-hand proteins were included in the network, all of which had ≥3 edges, and 5 of them had ≥6 edges. Proteins induced by SA treatment or *Cmm* infection alone interacted with those induced by both interventions to form clusters.

## Discussion

Several studies have demonstrated that the application of exogenous SA or its functional analogs can induce resistance to *Cmm*, suggesting that SA plays an important role in inducing immunity against tomato bacterial canker (Soylu et al. 2003; Yokotani et al. 2021). In this study, we identified SA-responsive genes via RNA-seq analysis and predicted the candidate genes involved in the immune response against *Cmm*. Results showed that various defense-associated genes, including PR and GST genes, were upregulated after SA treatment in tomato cotyledons (Table 1 and Supplementary Table S1). To the best of our knowledge, the genome-wide transcriptome of SA-treated tomato plants has not yet been elucidated, but the induction of PR and GST genes in SA-treated tomato cotyledons was similar to that previously reported in BTH-treated tomato (Zuluaga et al. 2013). A comparison of SA- and *Cmm*-responsive genes could help predict the important genes involved in immunity (Figure 1A). Similar over-represented GO terms were identified in SA- and *Cmm*-induced genes (Figure 1B, Supplementary Table S3). These findings suggested that the function and structure of proteins encoded by SA- and *Cmm*-induced genes showed a similar tendency. Studies have reported that many genes are upregulated after *Cmm* infection (Basim et al. 2021; Lara-Ávila et al. 2012; Yokotani et al. 2021). Among these, the genes that are also upregulated by SA treatment are suggested to be particularly important for inducing *Cmm* immunity. Furthermore, genes associated with defense signaling, including RGAs, Ca^2+^ sensors, and WRKY TFs, were commonly upregulated after both SA treatment and *Cmm* infection, suggesting their involvement in SA-mediated immune signaling against *Cmm*. They are encoded by a multigene family, and it remains unclear whether they function redundantly or diversely. The corresponding ligands of all SA-responsive RGAs have not yet been reported, and analysis of these RGAs could reveal novel aspects of plant immunity. Furthermore, ≥50% of the *Cmm*-responsive genes were not altered by SA treatment (Figure 1A). Notably, not all upregulated genes after *Cmm* infection were regulated by SA; this is because some genes should be affected by disease symptoms and others responded to MAMPs/PAMPs and bacterial effectors.

A total of 346 and 525 genes were downregulated after SA treatment and *Cmm* infection, respectively, with only 65 genes being commonly repressed after both interventions (Figure 1A). Previous studies have reported that *Cmm* infection suppresses photosynthesis-related gene expression in tomato, which may be associated with immunity (Tsitsekian et al. 2021; Yokotani et al. 2021), consistent with the results of the present study. However, the results of GO and KEGG enrichment analyses indicated that SA is not involved in the repression of photosynthesis-related gene expression (Supplementary Table S4). The reduced expression of photosynthesis-related genes in tomato after *Cmm* infection may be the result of yellowing, one of the disease symptoms. The results should therefore be carefully considered because the analysis times after SA treatment and *Cmm* infection were different. The only GO term and KEGG pathway commonly over-represented in genes downregulated after both SA treatment and *Cmm* infection were G1/S transition of the mitotic cell cycle (GO:0000082) and cyclin D3, plant (K14505), respectively, which are related to cell division (Supplementary Table S4). SA is known to suppress plant growth (Falcioni et al. 2014). It might play a role in growth and immunity trade-off during the interaction between tomato and *Cmm* by suppressing the expression of genes associated with cell division.

After SA treatment or *Cmm* infection, RGAs including RLKs, NBSs, RLPs, and TM-CCs were transcriptionally upregulated (Table 1). RLKs play important roles in PTI through the reception and mediation of MAMP signaling (Saijo et al. 2018; Tang et al. 2017). Although various types of RLKs, such as LRRs, WAKs, and RLCKs, have been reported to be upregulated by SA treatment, no RLK function has been reported in tomato to date (Sakamoto et al. 2012), suggesting that these proteins play unknown roles in immunity. In the current study, numerous non-RLK RGAs, including NBSs and RLPs, exhibited an increased expression and were over-represented in genes that were upregulated in both SA-treated and *Cmm*-infected cotyledons (Table 1). The non-RLK RGAs identified in this study are not likely to be involved in ETI because the host plant used in this study was a susceptible tomato cultivar. Several studies have revealed that non-RLK RGAs may be involved in disease resistance via mechanisms other than ETI. The soybean gene GmTNL16 was shown to be upregulated during infection and conferred resistance to *Phytophthora* root rot under overexpression conditions (Zhou et al. 2022). In *Arabidopsis*, the overexpression of *ADR1* increases resistance to virulent strains of fungal pathogens (Grant et al. 2003). In the abovementioned reports, the overexpression of RGAs in susceptible plants enhanced resistance, suggesting that their role in immunity is not limited to effector–R protein interaction. Similarly, SA- and *Cmm*-responsive RGAs may be involved in the regulation of plant immunity by acting differently from ETI.

In both SA- and *Cmm*-responsive genes, the GO term calcium ion binding (GO:0005509) was significantly over-represented. Intracellular Ca^2+^ levels are known to increase in infected plant cells, and various Ca^2+^ sensors are considered to play a role in immunity by recognizing this increase as secondary signaling (Dodd et al. 2010; Yang and Poovaiah 2003). Genes encoding calreticulin and EF-hand proteins were significantly over-represented in both SA- and *Cmm-*responsive genes. Calreticulin regulates Ca^2+^ homeostasis and protein folding in the endoplasmic reticulum and is associated with disease and abiotic stress response in plants (Christensen et al. 2008; Qiu et al. 2012). In this study, five calreticulin genes were detected in the tomato genome, two and three of which were induced by SA and *Cmm*, respectively, suggesting that calreticulin proteins play an important role in inducing immunity against *Cmm*. The EF-hand proteins transcriptionally induced by SA treatment or *Cmm* infection included calmodulins, calmodulin-like proteins, and CDPKs. Among these, CDPKs regulate the immune response by phosphorylating downstream target proteins, such as RBOHs, which are involved in the formation of hydrogen peroxide (Bredow and Monaghan 2019). Furthermore, genes encoding calmodulin and calmodulin-like proteins are induced by stress and disease and have been reported to be involved in defense responses (Snedden and Fromm 1998).

TFs, which directly regulate the expression of defense-associated genes, play an important role in plant immunity (Tsuda and Somssich 2015). In this study, three TF families, i.e., WRKYs, HSFs, and CBP60s, were over-represented in SA- or *Cmm*-responsive genes. The WRKY family is widely conserved in higher plants and is involved in the W-box-mediated expression of defense-associated genes (Ruston et al. 2010). Members of Groups I and III, which were induced after SA treatment and *Cmm* infection, are considered important for the disease response (Aamir et al. 2019; Huang et al. 2016). Furthermore, the W-box element, which is targeted by WRKY, was over-represented in the promoter region of genes that were upregulated after SA treatment or *Cmm* infection (Figure 2), suggesting that WRKY plays an important role in SA-mediated immunity against *Cmm*. HSF genes were also induced after SA treatment, implying that the response may be partly related to redox regulation (von Koskull-Döring et al. 2007). However, the HSE sequence used in this study (GAANNTTC) was not over-represented in the promoter region of SA-responsive genes, suggesting that other *cis*-elements in the defense-associated genes are involved in HSF-mediated regulation. CBP60s, a family of calmodulin-binding domain-containing proteins, regulate the expression of PR genes and SA biosynthesis in *Arabidopsis* (Sun et al. 2015). In this study, four CBP60 genes were upregulated after *Cmm* infection, but only one CBP60 gene was upregulated after SA treatment. The CBP60-targeted GAAATTT element was over-represented only in *Cmm*-responsive genes, in line with the expression patterns of CBP60 genes, which may function primarily in SA synthesis and not contribute significantly to SA downstream signaling. Another plant hormone, jasmonate (JA), is involved in plant immunity (Robert-Seilaniantz et al. 2011). In this study, few members of the ERF TFs, which are involved in JA-responsive gene regulation (Aerts et al. 2021), were transcriptionally altered by SA treatment or *Cmm* infection (Supplementary Tables S3 and S4). The transcript of SlMYC2, a crucial TF in the JA response (Robert-Seilaniantz et al. 2011), was also not altered by SA treatment or *Cmm* infection (Supplementary Tables S3 and S4). Therefore, JA may not be involved in the *Cmm* response. Consistent with these findings, our previous study demonstrated that the JA level in tomato cotyledon was not altered after *Cmm* infection (Yokotani et al. 2021).

Protein–protein interactions play an important role in signal transduction, and an analysis of their interactome network based on the PTIR database predicted that proteins encoded by SA- and *Cmm*-responsive genes interact with each other to form clusters (Figure 3, Supplementary Figure S2, Supplementary Table S5). The protein with the highest number of edges was HSP70, encoded by Solyc10g086410, but its interaction with other proteins may not be related to its specific role in immunity, as this protein functions as a molecular chaperone to protect protein folding (Cronjé and Bornman 1999). Further, several RLKs and EF-hand proteins contained a large number of edges. Some RLKs were shown to be specifically induced by SA, whereas others were induced by *Cmm*, suggesting their role in bridging SA and *Cmm* signaling to complete the immune response. Notably, interactions that are not included in the PTIR database are also involved in immunity. For example, CBP60 may be associated with calmodulin (Sun et al. 2015). In addition, proteins that do not require de novo transcription may be included in the interactions. Thus, the interactome network is likely to be much more complex than anticipated in this study.

After SA treatment, several genes were altered in tomato cotyledons. Genes that were upregulated by SA treatment contained various immune-associated genes and also responded to *Cmm* infection. Furthermore, GO and KEGG enrichment analyses revealed that genes that were upregulated after both SA treatment and *Cmm* infection had similar functions. Our study indicated that PR genes, GST genes, RGA, Ca^2+^ sensors, and WRKY TFs were over-represented in the genes upregulated after both interventions. The W-box, targeted by WRKY TFs, was over-represented in the promoter region of genes upregulated by SA treatment or *Cmm* infection, supporting the speculation that the WRKY family is essential for the SA-mediated immunity against *Cmm*. The prediction of protein–protein interactions encoded by genes upregulated by SA treatment or *Cmm* infection suggested that RLK and EF-hand proteins play important roles in immune signaling. RGAs such as RLKs and NBSs have been reported to improve disease resistance in transgenic plants through their overexpression (Grant et al. 2003; Tang et al. 2017). They are considered a target for enhancing plant disease resistance through genome editing (Andolfo et al. 2016). Our findings identified candidate genes responsible for SA-inducible resistance to *Cmm*, which may be targeted in molecular breeding programs to improve disease resistance in tomato.

## Abbreviations

*Cmm*: *Clavibacter michiganensis* ssp. *michiganensis*
PPIs: protein–protein interactions
RGA: resistance gene analog
RLK: receptor-like kinase
SA: salicylic acid
TF: transcription factor

## Description of Supplementary Files

Supplementary Figure S1. Heatmap of genes altered after SA treatment or *Cmm* infection.

Supplementary Figure S2. Protein–protein interactome network of all genes upregulated after SA treatment or *Cmm* infection.

Supplementary Table S1. Read counts and mapping status of sequencing data.

Supplementary Table S2. Mean CPM values of the tomato cotyledons.

Supplementary Table S3. GO and KEGG enrichment analyses of genes upregulated after SA treatment or *Cmm* infection.

Supplementary Table S4. GO and KEGG enrichment analyses of genes downregulated after SA treatment or *Cmm* infection.

Supplementary Table S5. List of genes used in PPI.

## References

[RAamir2019] Aamir M, Kashyap SP, Zehra A, Dubey MK, Singh VK, Ansari WA, Upadhyay RS, Singh S (2019) *Trichoderma erinaceum* bio-priming modulates the WRKYs defense programming in tomato against the *Fusarium oxysporum* f. sp. *lycopersici* (Fol) challenged condition. *Front Plant Sci* 10: 911 31428107 10.3389/fpls.2019.00911 PMC6689972

[RAerts2021] Aerts N, Pereira Mendes M, Van Wees SCM (2021) Multiple levels of crosstalk in hormone networks regulating plant defense. *Plant J* 105: 489–504 33617121 10.1111/tpj.15124 PMC7898868

[RAndolfo2016] Andolfo G, Iovieno P, Frusciante L, Ercolano MR (2016) Genome-editing technologies for enhancing plant disease resistance. *Front Plant Sci* 7: 1813 27990151 10.3389/fpls.2016.01813 PMC5130979

[RBalaji2008] Balaji V, Mayrose M, Sherf O, Jacob-Hirsch J, Eichenlaub R, Iraki N, Manulis-Sasson S, Rechavi G, Barash I, Sessa G (2008) Tomato transcriptional changes in response to *Clavibacter michiganensis* subsp. *michiganensis* reveal a role for ethylene in disease development. *Plant Physiol* 146: 1797–1809 18245454 10.1104/pp.107.115188 PMC2287351

[RBasim2021] Basim H, Basim E, Tombuloglu H, Unver T (2021) Comparative transcriptome analysis of resistant and cultivated tomato lines in response to *Clavibacter michiganensis* subsp. *michiganensis.* *Genomics* 113: 2455–2467 34052318 10.1016/j.ygeno.2021.05.033

[RBredow2019] Bredow M, Monaghan J (2019) Regulation of plant immune signaling by calcium-dependent protein kinases. *Mol Plant Microbe Interact* 32: 6–19 30299213 10.1094/MPMI-09-18-0267-FI

[RBuchfink2015] Buchfink B, Xie C, Huson DH (2015) Fast and sensitive protein alignment using DIAMOND. *Nat Methods* 12: 59–60 25402007 10.1038/nmeth.3176

[RBusch2005] Busch W, Wunderlich M, Schöffl F (2005) Identification of novel heat shock factor-dependent genes and biochemical pathways in *Arabidopsis thaliana.* *Plant J* 41: 1–14 15610345 10.1111/j.1365-313X.2004.02272.x

[RChristensen2008] Christensen A, Svensson K, Persson S, Jung J, Michalak M, Widell S, Sommarin M (2008) Functional characterization of Arabidopsis calreticulin1a: A key alleviator of endoplasmic reticulum stress. *Plant Cell Physiol* 49: 912–924 18436549 10.1093/pcp/pcn065

[RConesa2005] Conesa A, Götz S, García-Gómez JM, Terol J, Talón M, Robles M (2005) Blast2GO: A universal tool for annotation, visualization and analysis in functional genomics research. *Bioinformatics* 21: 3674–3676 16081474 10.1093/bioinformatics/bti610

[d66e1489] Cronjé MJ, Bornman L (1999) Salicylic acid influences Hsp70/Hsc70 expression in *Lycopersicon esculentum*: Dose- and time-dependent induction or potentiation. *Biochem Biophys Res Commun* 265: 422–427 10558883 10.1006/bbrc.1999.1692

[RDodd2010] Dodd AN, Kudla J, Sanders D (2010) The language of calcium signaling. *Annu Rev Plant Biol* 61: 593–620 20192754 10.1146/annurev-arplant-070109-104628

[REichenlaub2011] Eichenlaub R, Gartemann KH (2011) The *Clavibacter michiganensis* subspecies: Molecular investigation of gram-positive bacterial plant pathogens. *Annu Rev Phytopathol* 49: 445–464 21438679 10.1146/annurev-phyto-072910-095258

[RFalcioni2014] Falcioni T, Ferrio JP, del Cueto AI, Giné J, Achón MÁ, Medina V (2014) Effect of salicylic acid treatment on tomato plant physiology and tolerance to potato virus X infection. *Eur J Plant Pathol* 138: 331–345

[RFinn2011] Finn RD, Clements J, Eddy SR (2011) HMMER web server: Interactive sequence similarity searching. *Nucleic Acids Res* 39(suppl): W29–W37 21593126 10.1093/nar/gkr367 PMC3125773

[RGrant2003] Grant JJ, Chini A, Basu D, Loake GJ (2003) Targeted activation tagging of the Arabidopsis NBS-LRR gene, ADR1, conveys resistance to virulent pathogens. *Mol Plant Microbe Interact* 16: 669–680 12906111 10.1094/MPMI.2003.16.8.669

[RHou2019] Hou S, Liu Z, Shen H, Wu D (2019) Damage-associated molecular pattern-triggered immunity in plants. *Front Plant Sci* 10: 646 31191574 10.3389/fpls.2019.00646 PMC6547358

[RHuang2016] Huang Y, Li MY, Wu P, Xu ZS, Que F, Wang F, Xiong AS (2016) Members of WRKY Group III transcription factors are important in TYLCV defense signaling pathway in tomato (*Solanum lycopersicum*). *BMC Genomics* 17: 788 27717312 10.1186/s12864-016-3123-2 PMC5055730

[RKudo2017] Kudo T, Kobayashi M, Terashima S, Katayama M, Ozaki S, Kanno M, Saito M, Yokoyama K, Ohyanagi H, Aoki K, et al. (2017) TOMATOMICS: A web database for integrated omics information in tomato. *Plant Cell Physiol* 58: e8 28111364 10.1093/pcp/pcw207 PMC5444566

[RKusajima2017] Kusajima M, Okumura Y, Fujita M, Nakashita H (2017) Abscisic acid modulates salicylic acid biosynthesis for systemic acquired resistance in tomato. *Biosci Biotechnol Biochem* 81: 1850–1853 28673127 10.1080/09168451.2017.1343121

[RLara2012] Lara-Ávila JP, Isordia-Jasso MI, Castillo-Collazo R, Simpson J, Alpuche-Solis ÁG (2012) Gene expression analysis during interaction of tomato and related wild species with *Clavibacter michiganensis* subsp. *michiganensis.* *Plant Mol Biol Report* 30: 498–511

[RLi2016] Li P, Quan X, Jia G, Xiao J, Cloutier S, You FM (2016) RGAugury: A pipeline for genome-wide prediction of resistance gene analogs (RGAs) in plants. *BMC Genomics* 17: 852 27806688 10.1186/s12864-016-3197-x PMC5093994

[RMaleck2000] Maleck K, Levine A, Eulgem T, Morgan A, Schmid J, Lawton KA, Dangl JL, Dietrich RA (2000) The transcriptome of *Arabidopsis thaliana* during systemic acquired resistance. *Nat Genet* 26: 403–410 11101835 10.1038/82521

[RMenda2013] Menda N, Strickler SR, Mueller LA (2013) Advances in tomato research in the post-genome era. *Plant Biotechnol* 30: 243–256

[RMistry2021] Mistry J, Chuguransky S, Williams L, Qureshi M, Salazar GA, Sonnhammer ELL, Tosatto SCE, Paladin L, Raj S, Richardson LJ, et al. (2021) Pfam: The protein families database in 2021. *Nucleic Acids Res* 49(D1): D412–D419 33125078 10.1093/nar/gkaa913 PMC7779014

[RMunir2016] Munir S, Khan MRG, Song J, Munir S, Zhang Y, Ye Z, Wang T (2016) Genome-wide identification, characterization and expression analysis of calmodulin-like (CML) proteins in tomato (*Solanum lycopersicum*). *Plant Physiol Biochem* 102: 167–179 26949025 10.1016/j.plaphy.2016.02.020

[RQiu2012] Qiu Y, Xi J, Du L, Roje S, Poovaiah BW (2012) A dual regulatory role of Arabidopsis calreticulin-2 in plant innate immunity. *Plant J* 69: 489–500 21974727 10.1111/j.1365-313X.2011.04807.x

[RRobert2011] Robert-Seilaniantz A, Grant M, Jones JD (2011) Hormone crosstalk in plant disease and defense: More than just jasmonate-salicylate antagonism. *Annu Rev Phytopathol* 49: 317–343 21663438 10.1146/annurev-phyto-073009-114447

[RRuston2010] Ruston PJ, Somssich IE, Ringler P, Shen QJ (2010) WRKY transcription factors. *Trends Plant Sci* 15: 247–258 20304701 10.1016/j.tplants.2010.02.006

[RSaijo2018] Saijo Y, Loo EP, Yasuda S (2018) Pattern recognition receptors and signaling in plant-microbe interactions. *Plant J* 93: 592–613 29266555 10.1111/tpj.13808

[RSakamoto2012] Sakamoto T, Deguchi M, Brustolini OJB, Santos AA, Silva FF, Fontes EPB (2012) The tomato RLK superfamily: Phylogeny and functional predictions about the role of the LRRII-RLK subfamily in antiviral defense. *BMC Plant Biol* 12: 229 23198823 10.1186/1471-2229-12-229 PMC3552996

[RSchwessinger2012] Schwessinger B, Ronald PC (2012) Plant innate immunity: Perception of conserved microbial signatures. *Annu Rev Plant Biol* 63: 451–482 22404464 10.1146/annurev-arplant-042811-105518

[RSen2015] Sen Y, van der Wolf J, Visser RGF, van Heusden S (2015) Bacterial canker of tomato: Current knowledge of detection, management, resistance, and interactions. *Plant Dis* 99: 4–13 30699746 10.1094/PDIS-05-14-0499-FE

[RSnedden1998] Snedden WA, Fromm H (1998) Calmodulin, calmodulin-related proteins and plant responses to the environment. *Trends Plant Sci* 3: 299–304

[RSoylu2003] Soylu S, Baysal Ö, Soylu EM (2003) Induction of disease resistance by the plant activator, acibenzolar-S-methyl (ASM), against bacterial canker (*Clavibacter michiganensis* subsp. *michiganensis*) in tomato seedlings. *Plant Sci* 165: 1069–1075

[RSun2015] Sun T, Zhang Y, Li Y, Zhang Q, Ding Y, Zhang Y (2015) ChIP-seq reveals broad roles of SARD1 and CBP60g in regulating plant immunity. *Nat Commun* 6: 10159 27206545 10.1038/ncomms10159 PMC4703862

[RTang2017] Tang D, Wang G, Zhou JM (2017) Receptor kinases in plant-pathogen interactions: More than pattern recognition. *Plant Cell* 29: 618–637 28302675 10.1105/tpc.16.00891 PMC5435430

[RTena2011] Tena G, Boudsocq M, Sheen J (2011) Protein kinase signaling networks in plant innate immunity. *Curr Opin Plant Biol* 14: 519–529 21704551 10.1016/j.pbi.2011.05.006 PMC3191242

[RTomato2012] Tomato Genome Consortium (2012) The tomato genome sequence provides insights into fleshy fruit evolution. *Nature* 485: 635–641 22660326 10.1038/nature11119 PMC3378239

[RTsitsekian2021] Tsitsekian D, Daras G, Karamanou K, Templalexis D, Koudounas K, Malliarakis D, Koufakis T, Chatzopoulos D, Goumas D, Ntoukakis V, et al. (2021) *Clavibacter michiganensis* downregulates photosynthesis and modifies monolignols metabolism revealing a crosstalk with tomato immune responses. *Int J Mol Sci* 22: 8442 34445148 10.3390/ijms22168442 PMC8395114

[RTsuda2015] Tsuda K, Somssich IE (2015) Transcriptional networks in plant immunity. *New Phytol* 206: 932–947 25623163 10.1111/nph.13286

[Rvan2006] van Loon LC, Rep M, Pieterse CM (2006) Significance of inducible defense-related proteins in infected plants. *Annu Rev Phytopathol* 44: 135–162 16602946 10.1146/annurev.phyto.44.070505.143425

[Rvon2007] von Koskull-Döring P, Scharf KD, Nover L (2007) The diversity of plant heat stress transcription factors. *Trends Plant Sci* 12: 452–457 17826296 10.1016/j.tplants.2007.08.014

[RWang2016] Wang JP, Xu YP, Munyampundu JP, Liu TY, Cai XZ (2016) Calcium-dependent protein kinase (CDPK) and CDPK-related kinase (CRK) gene families in tomato: Genome-wide identification and functional analyses in disease resistance. *Mol Genet Genomics* 291: 661–676 26520101 10.1007/s00438-015-1137-0

[RYang2003] Yang T, Poovaiah BW (2003) Calcium/calmodulin-mediated signal network in plants. *Trends Plant Sci* 8: 505–512 14557048 10.1016/j.tplants.2003.09.004

[RYokotani2021] Yokotani N, Hasegawa Y, Sato M, Hirakawa H, Kouzai Y, Nishizawa Y, Yamamoto E, Naito Y, Isobe S (2021) Transcriptome analysis of *Clavibacter michiganensis* subsp. *michiganensis*-infected tomatoes: A role of salicylic acid in the host response. *BMC Plant Biol* 21: 476 34666675 10.1186/s12870-021-03251-8 PMC8524973

[RYue2016] Yue J, Xu W, Ban R, Huang S, Miao M, Tang X, Liu G, Liu Y (2016) PTIR: Predicted tomato interactome resource. *Sci Rep* 6: 25047 27121261 10.1038/srep25047 PMC4848565

[RZhao2013] Zhao Y, Liu W, Xu YP, Cao JY, Braam J, Cai XZ (2013) Genome-wide identification and functional analyses of calmodulin genes in *Solanaceous* species. *BMC Plant Biol* 13: 70 23621884 10.1186/1471-2229-13-70 PMC3751459

[RZhou2020] Zhou JM, Zhang Y (2020) Plant immunity: Danger perception and signaling. *Cell* 181: 978–989 32442407 10.1016/j.cell.2020.04.028

[RZhou2022] Zhou L, Deng S, Xuan H, Fan X, Sun R, Zhao J, Wang H, Guo N, Xing H (2022) A novel TIR-NBS-LRR gene regulates immune response to Phytophthora root rot in soybean. *Crop J* 10: 1644–1653

[RZuluaga2013] Zuluaga AP, Vega-Arreguín JC, Fry WE (2013) Transcriptome profile of acibenzolar-S-methyl-induced genes in tomato suggests a complex polygenic effect on resistance to *Phytophthora infestans.* *Physiol Mol Plant Pathol* 81: 97–106

