# Supplementary material for: Transcriptome analysis of tomato plants following salicylic acid-induced immunity against Clavibacter michiganensis ssp. michiganensis
Source: Plant Biotechnol (Tokyo). 2023 Dec 25;40(4):273–82. doi: 10.5511/plantbiotechnology.23.0711a (PMC10905565; doi:10.5511/plantbiotechnology.23.0711a)
Supplement: Supplementary Data [file plantbiotechnology-40-4-23.0711a-s002.pdf]

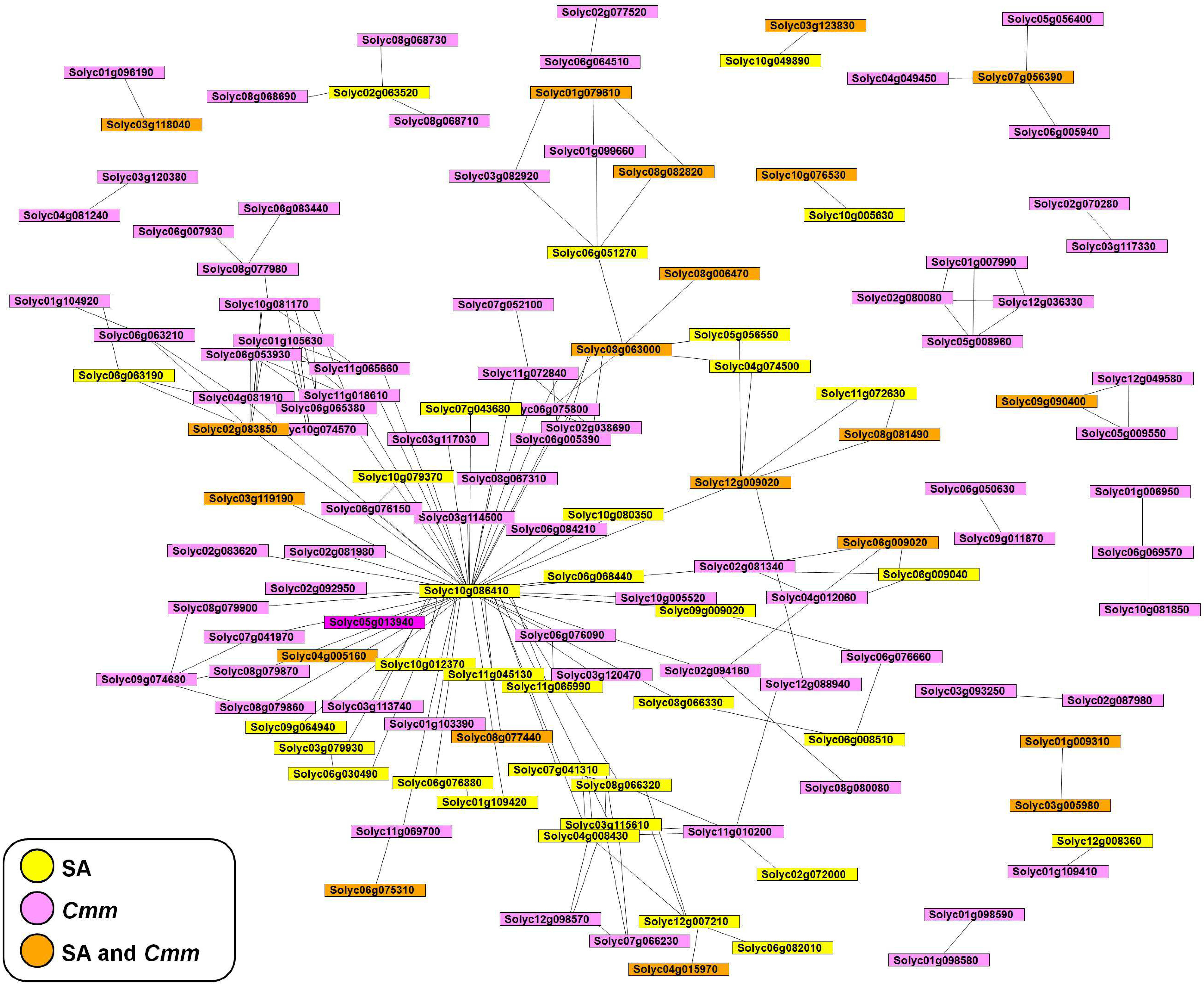

Supplementary Figure S2. Protein−protein interactome network of all genes upregulated after SA treatment or *Cmm* infection.
